# Functional and Molecular Characterization of the Role of CTCF in Human Embryonic Stem Cell Biology

**DOI:** 10.1371/journal.pone.0042424

**Published:** 2012-08-03

**Authors:** Sri Kripa Balakrishnan, Michael Witcher, Travis W. Berggren, Beverly M. Emerson

**Affiliations:** 1 Regulatory Biology Laboratory, Salk Institute for Biological Studies, La Jolla, California, United States of America; 2 Stem Cell Core, Salk Institute for Biological Studies, La Jolla, California, United States of America; National Institutes of Health, United States of America

## Abstract

The CCCTC-binding factor CTCF is the only known vertebrate insulator protein and has been shown to regulate important developmental processes such as imprinting, X-chromosome inactivation and genomic architecture. In this study, we examined the role of CTCF in human embryonic stem cell (hESC) biology. We demonstrate that CTCF associates with several important pluripotency genes, including *NANOG, SOX2*, *cMYC* and *LIN28* and is critical for hESC proliferation. CTCF depletion impacts expression of pluripotency genes and accelerates loss of pluripotency upon BMP4 induced differentiation, but does not result in spontaneous differentiation. We find that CTCF associates with the distal ends and internal sites of the co-regulated 160 kb *NANOG-DPPA3-GDF3* locus. Each of these sites can function as a CTCF-dependent enhancer-blocking insulator in heterologous assays. In hESCs, CTCF exists in multisubunit protein complexes and can be poly(ADP)ribosylated. Known CTCF cofactors, such as Cohesin, differentially co-localize in the vicinity of specific CTCF binding sites within the *NANOG* locus. Importantly, the association of some cofactors and protein PARlation selectively changes upon differentiation although CTCF binding remains constant. Understanding how unique cofactors may impart specialized functions to CTCF at specific genomic locations will further illuminate its role in stem cell biology.

## Introduction

Embryonic stem cells (ESCs) are derived from blastocysts and are considered pluripotent since they have the potential to give rise to a myriad of cell types. For this reason they are of great therapeutic value. However, before stem cells or induced pluripotent stem (iPS) cells are taken to the clinic, a greater understanding of the basic biology of embryonic stem cells is needed. Embryonic stem cells have the ability to self-renew and proliferate indefinitely in culture and several studies have described the importance of the core regulatory circuitry that is comprised of NANOG, OCT4 and SOX2 proteins to maintain a pluripotent state [1], [2]. Besides these, other proteins such as cMYC, KLF4 and LIN28 are critical not only to maintain stemness but also to induce pluripotency from differentiated cells (reviewed in [3]). Furthermore, gene knockdown experiments in both mouse and human ESCs have shown that core transcriptional regulatory proteins such as subunits of the Mediator complex are important for activation of pluripotency genes such as *OCT4* and *NANOG* [4], [5].

Proteins like NANOG, OCT4 and SOX2 regulate gene expression in a classic manner, by binding to target gene promoters and facilitating transcriptional activation. Other components, such as insulator proteins, function differently from canonical transcription factors by segregating genomic loci into distinct domains to protect them from inappropriate activation or repression by adjacent DNA regions (reviewed in [6]). Importantly, the CCCTC-binding factor CTCF is the only known vertebrate insulator protein. CTCF can function either as an enhancer-blocking or barrier insulator. Enhancer-blocking insulators prevent improper communication between regulatory elements such as enhancers and promoters, while barrier insulators prevent inappropriate spreading of heterochromatin from neighboring domains. CTCF is also important in key developmental processes such as genomic imprinting and X-chromosome inactivation (reviewed [7]). In addition, CTCF knockout mice are embryonic lethal and depletion of CTCF from oocytes results in misregulation of gene expression programs accompanied by meiotic defects [8], [9].

At the molecular level, episomal enhancer-blocking assays have led to the identification of several enhancer-blocking insulators including the 5′ hypersensitive site-4 site (5′HS-4) from the chicken β-globin locus and the *H19/IGF2* Imprinted Control Region (ICR) [10]–[12]. CTCF binding to several sites within the maternal ICR blocks intra-chromosomal communication between downstream enhancers and the *IGF2* promoter, thus silencing *IGF2* on the maternal allele. However, on the paternal allele, CTCF binding is abrogated due to methylation of ICR thereby rendering *IGF2* transcriptionally active. Furthermore, deletion of CTCF binding sites within this locus results in loss of enhancer-blocking activity [10], [12]–[15]. Interestingly, genome-wide binding studies have shown that at a small subset of genes, CTCF can separate active (H2AK5Ac-enriched) and repressive (H3K27Me3-enriched) domains in a cell-type specific manner suggesting that it has a barrier insulator function at these genes [16]. In addition, a critical CTCF-dependent chromatin boundary has been identified upstream of the transcriptionally active *p16* tumor suppressor gene, which segregates it from an adjacent region of heterochromatin. Intriguingly, aberrant epigenetic silencing of the *p16* gene, which is widespread among human cancers, occurs when the boundary destabilizes upon loss of CTCF binding, and nearby heterochromatin spreads into the locus [17].

Genome-wide CTCF binding analyses indicate that CTCF association with DNA is largely conserved across cell types including pluripotent and differentiated hESCs, even though gene expression patterns differ considerably among distinct tissues and species [18], [19]. These data provide information on genome-wide CTCF localization, however, the functional significance of CTCF binding and its lack of variance across cell types is not clear. Furthermore, the importance of CTCF in hESC biology and the role of insulator elements, given the unique chromatin structure in pluripotent cells, have not yet been described.

In this study, we sought to bridge this gap. Our data show that depletion of CTCF in hESCs does not lead to spontaneous differentiation of hESCs although hESC proliferation and expression of certain genes implicated in pluripotency regulation, such as *NANOG, SOX2*, *cMYC*, *KLF4* and *LIN28,* are affected. CTCF-depletion accelerates BMP4-induced loss of pluripotency. We find that CTCF associates with the distal ends of the co-regulated 160 kb *NANOG-DPPA3-GDF3* locus in hESCs and that each of these sites can serve as CTCF-dependent enhancer-blocking insulator in a heterologous assay. Interestingly, CTCF binding to the *NANOG* locus does not change upon BMP4-induced differentiation, however the interaction of CTCF cofactors is selectively modulated at particular CTCF-bound sites.

## Results

### Characterization of the hESC Model

**Figure 1:**
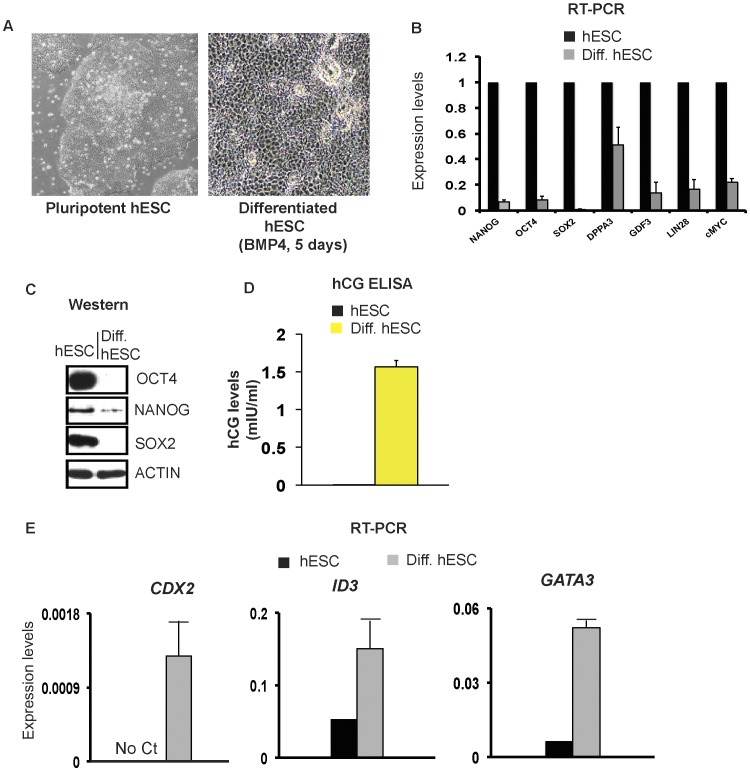
Characterization of the hESC differentiation model. A. 10X Phase contrast images of H9 pluripotent hESCs and H9 hESCs induced to differentiate with BMP4 for 5 days. 200 ng/ml BMP4 in TeSR was used in all panels. B. RT-PCR analysis showing decrease in mRNA levels of indicated genes upon BMP4 treatment of H9 cells. Expression levels are normalized to *GAPDH* and represented relative to pluripotent ESCs set to 1. C. Western analysis showing decrease in expression levels of indicated proteins upon BMP4 treatment of H9 cells. D. hCG ELISA from supernatant of BMP4-treated (yellow bars) and control-treated H9 (black bars hCG levels in control cells (hESCs) were barely detectable. E. RT-PCR analysis showing increase in mRNA levels of indicated genes upon BMP4 treatment of H9. Expression levels are normalized to *GAPDH*.

To investigate the role of CTCF in hESCs, we used two model systems: (1) hESCs grown under defined conditions, i.e, in Matrigel and TeSR (pluripotent hESCs) and (2) hESCs treated with BMP4 to induce differentiation (differentiated hESCs) [20]. BMP4 treatment results in complete loss of hESC pluripotency and directs cells homogeneously towards a trophoblast lineage [21]. We confirmed that treatment of hESCs with BMP4 for 5 days results in profound morphological changes characterized by loss of defined colonies, formation of flattened cells and a decreased nuclear-cytoplasmic ratio (Figure 1A). Concomitantly, BMP4 treatment decreases mRNA levels of pluripotency genes such as *NANOG*, *OCT4*, *SOX2*, *DPPA3*, *GDF3, LIN28* and *cMYC* while increasing expression of a BMP4-responsive gene-*ID3* (Figures 1B and 1E) [22]–[24]. Moreover, we show that loss of expression of key pluripotent genes is recapitulated at the protein level (Figures 1C). Finally, at high concentrations of BMP4 (200 ng/ml), we observed an increase in the human Chorionic Gonadotrophic hormone (hCG) and up-regulation of trophoblast markers such as *CDX2* and *GATA3* indicating that these cells are committed towards the trophoblast lineage (Figures 1D and E) [25], [26]. These observations validate that *in vitro* differentiation of hESCs with BMP4 results in loss of self-renewal with commitment towards the trophoblast lineage. This model of hESC differentiation has been utilized in other studies [19], [27].

### CTCF Depletion Affects Expression of Genes Implicated in Pluripotency

**Figure 2:**
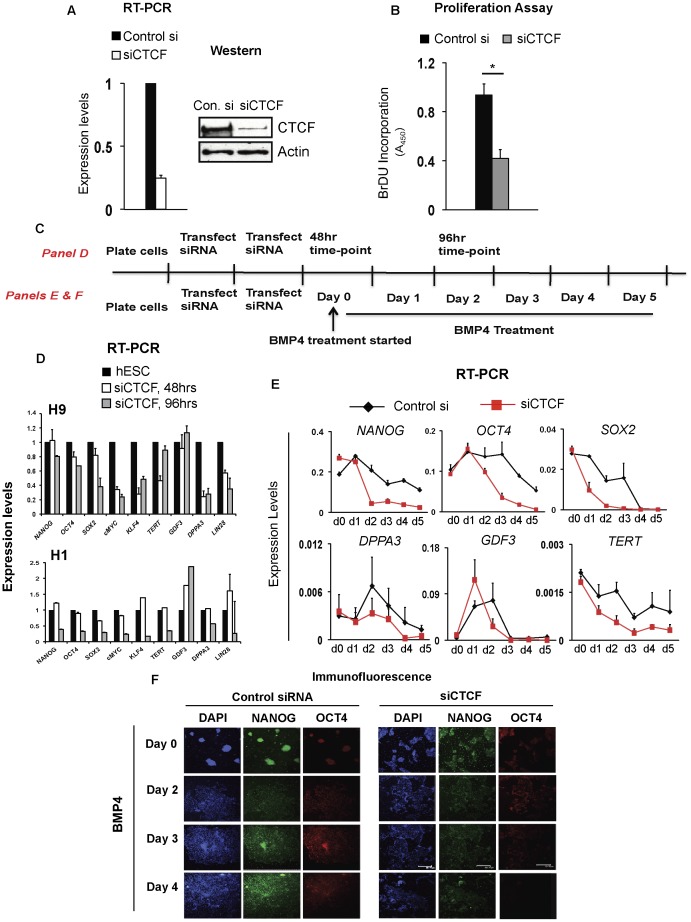
Effect of CTCF depletion on hESCs. A. RT-PCR (left) and Western analysis (right) showing CTCF mRNA and protein levels, respectively, in H9 hESCs transfected with a scrambled siRNA (control si) or siCTCF. mRNA levels are normalized to *GAPDH* and represented relative to pluripotent hESC set to 1. B. BrDU proliferation assay in H9 showing that CTCF knockdown impairs proliferation. A_450_ for siCTCF is normalized to that of control si (scrambled control) set to 1. Mean ± SEM represented. * represents p<0.05. C. Schematic of experimental design for Figures 2D, E and F. Each vertical line represents a 24 hr period. D. mRNA levels of indicated genes at 48 hrs and 96 hrs after CTCF knockdown in H9 and H1 hESCs. mRNA levels of indicated genes in control si and siCTCF were normalized to respective *GAPDH* levels. Subsequently, mRNA levels of siCTCF were normalized to control si set to 1. Mean ± SEM represented. E. RT-PCR analysis of indicated genes upon control si (black line) or siCTCF (red line) transfection followed by BMP4 treatment for 5 days in H9. mRNA levels were analyzed at the indicated days and normalized to *GAPDH*. Mean ± SEM represented. F. Immunofluorescence of NANOG and OCT4 proteins upon control si or siCTCF transfection followed by BMP4 treatment at indicated days in H9. Scale bar represents 360 µm.

Genome-wide CTCF binding profiles have been elucidated in a variety of cell types including mouse and human ESCs; however, the significance of CTCF association with specific genomic regions remains unknown [19], [28]. To investigate the functional importance of CTCF in hESCs, we depleted CTCF using siRNA. Transfection of siRNA targeting CTCF results in near complete loss of CTCF expression at both mRNA and protein levels (Figures 2A and S1A). Next, we analyzed the effect of CTCF on hESC proliferation by measuring BrDU incorporation in cells depleted of CTCF. Notably, CTCF ablation resulted in 60% less BrDU incorporation compared to cells transfected with a scrambled control (Figure 2B). Decreased proliferation is also phenotypically recapitulated in the morphology of CTCF- depleted cells. These cells are characterized by loss of defined colonies and formation of thinner, elongated colonies (Figures S1B and S2B). These data support the notion that CTCF is critical for hESC proliferation. Importantly, changes in morphology and proliferation are not due to increased cell death in CTCF depleted cells as judged by propidium iodide staining (Figure S3).

Next, we asked if CTCF depletion affects expression of genes connected with pluripotency regulation. To this end, we measured transcript levels of several genes known to be important in hESCs upon CTCF ablation (experimental design, Figure 2C). Interestingly, CTCF depletion decreases expression levels of genes such as *NANOG*, *OCT4*, *SOX2, DPPA3* and *TERT* to different degrees (Figures 2D, S2C and S4). Moreover, other critical genes implicated in self-renewal and proliferation such as *cMYC*, *LIN28* and *KLF4* were strongly affected (>65% decrease) upon loss of CTCF. *cMYC* has been implicated in maintaining proliferation of ESCs; therefore loss of proliferation is consistent with decreased *cMYC* mRNA upon CTCF knockdown (Figures 2B and D). By contrast, CTCF ablation did not influence *GDF3* transcript levels in either cell line. Rather, *GDF3* was up-regulated two-fold in H1 hESCs transfected with siCTCF, suggesting that CTCF may have different roles in the regulation of stem cell genes. Interestingly, CTCF depletion in H1 hESCs had a more profound impact than in H9 hESCs.

### CTCF Depletion Accelerates Loss of Pluripotency Markers Upon Induction of Differentiation

Next, we asked if decreased CTCF levels accelerate differentiation of hESCs. To test this, we treated CTCF-depleted and control cells with BMP4 and monitored the mRNA levels of key pluripotency genes over a period of five days. BMP4 treatment following CTCF depletion results in an accelerated loss of pluripotency markers compared to BMP4-treated cells transfected with a scrambled control (compare red line to black line, Figure 2E). Differences in TERT expression at 48 hrs in figure 2D (for H9) and day 0 in figure 2E could be reflected by the differences in media components used in these experiments. Indeed, different media components can have subtle effects on differentiation [29]. Consistent with the mRNA levels, CTCF depletion also results in a more rapid loss of NANOG and OCT4 protein expression upon BMP4 stimulation (Figure 2F). In addition, we found an accelerated loss of pluripotency markers such as *NANOG* and *OCT4* in CTCF-depleted cells compared to control upon use of TPA (phorbol 12-myristate 13-acetate), which drives cells towards the epithelial mesenchymal lineage (Figure S5A) [30]. Similarly, upon treatment with retinoic acid, a known inducer of ESC differentiation, we found a greater decrease in *NANOG* expression in CTCF-depleted cells (Figure S5B). However, we observed only a subtle difference between CTCF-depleted and control cells when cells were differentiated to form embryoid bodies (Figure S5C). Overall, these observations show that the level of down-regulation of pluripotency genes upon loss of CTCF depends on the differentiation stimulus.

### CTCF Associates with the Critical *NANOG-DPPA3-GDF3* Locus

**Figure 3:**
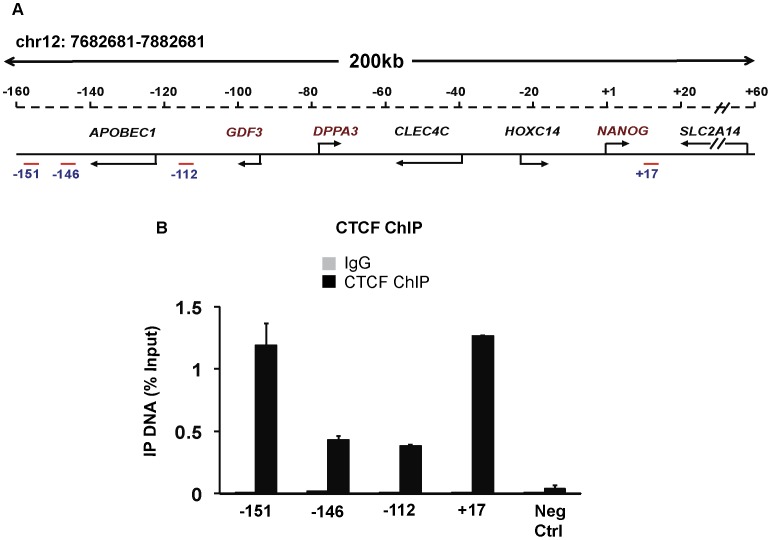
CTCF associates with the *NANOG-DPPA3-GDF3* locus in hESCs. A. Schematic of the human *NANOG* locus that encompasses the co-regulated *NANOG-DPPA3-GDF3* genes. Co-ordinates are represented relative to the *NANOG* transcription start site set to +1. Red amplicons represent PCR primers used for Chromatin Immunoprecipitation (ChIP) analyses. B. Chromatin Immunoprecipitation (ChIP)-qPCR analyses using α-IgG (control) and α-CTCF in H9 hESCs. Amplicons as in panel A. A PCR primer pair that amplifies a region within the *DPPA3* gene (−74 kb upstream of *NANOG* transcription start site) was used as a negative control. Immunoprecipitated DNA is represented as a percentage of input amplified with the same PCR primers. Mean ± SEM represented.

CTCF is a diverse protein that can function as an activator, repressor or insulator, thus we hypothesized that CTCF may associate with and regulate critical stem cell genes/gene loci. Genome-wide CTCF binding studies have been conducted across terminally differentiated cell types (Krawczyk and Emerson, unpublished observations; [31]); these studies identified CTCF association with known pluripotency genes. We tested CTCF interaction with genomic regions surrounding the *NANOG* locus in pluripotent H9 hESCs (referred to as the *NANOG* locus, Figure 3A) since the *NANOG* gene is considered to be a master regulator of the ES cell regulatory network [3]. Similar to data from other cell types, we detected CTCF enrichment in hESCs at four distinct sites flanking the *NANOG* locus: −151, −146, −112 and +17 kb relative to the *NANOG* transcription start site (Figure 3B). Intriguingly, this locus, spread over ∼160 kb on chr12p13, encompasses the co-regulated *NANOG*, *DPPA3* and *GDF3* genes (schematic diagram, Figure 3A). In addition, we examined the presence of CTCF consensus motifs at these four CTCF-bound regions using a software algorithm (http://insulatordb.uthsc.edu/) as described in [32]. As shown in Figure S6A, all four CTCF-bound sites contain at least one CTCF consensus motif that matches the consensus defined by Kim et al. [33]. While our study was in progress, global ChIP-seq analyses in H1 hESCs further validated that these four sites were bound by CTCF in hESCs ( [18] and data deposited on UCSC genome browser; Figure S6B).

### CTCF Binding Sites within the *NANOG* Locus can Function as Enhancer Blockers in a CTCF-Dependent Manner

**Figure 4:**
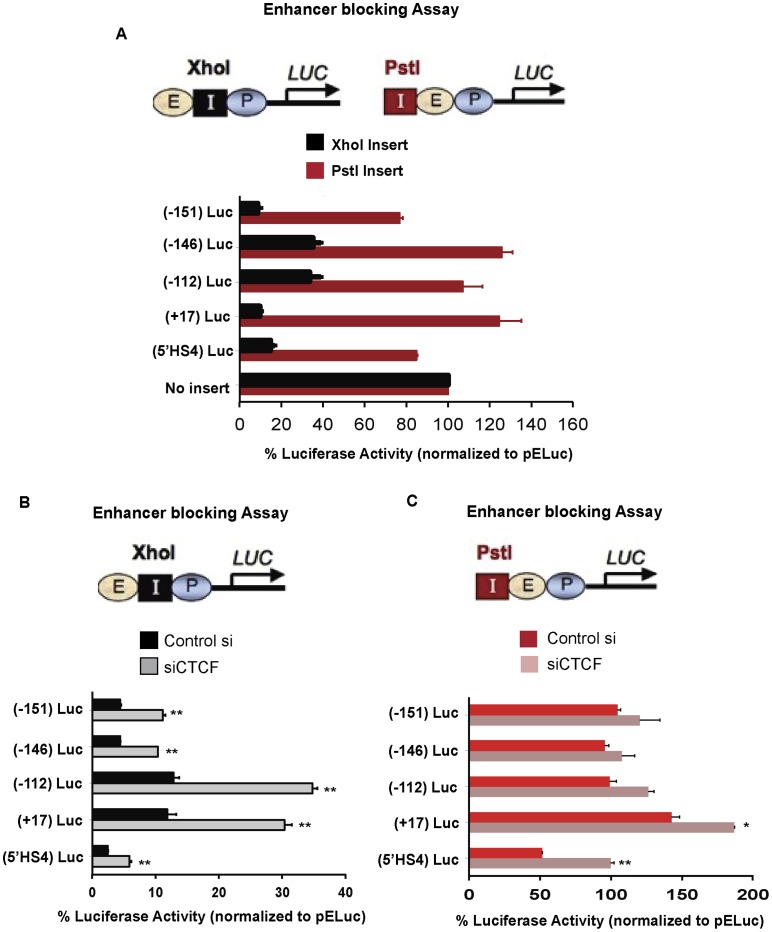
CTCF sites within the *NANOG* locus can function as CTCF-dependent enhancer-blocking insulators. A. Luciferase reporter assay measuring enhancer-blocking activity of CTCF sites from the *NANOG* locus. Enhancer-blocking plasmids contained a CMV enhancer, a CMV promoter-driven luciferase reporter, and 1.2 kb of DNA encompassing CTCF binding sites from relevant regions were cloned into either XhoI or Pst1 restriction enzyme sites. *Luciferase* activity was normalized to that of a co-transfected *renilla-luciferase* and normalized relative to pELuc (empty vector) set to 100%. 5′HS4 from the chicken β-globin locus was used as a positive control. Mean ± SEM represented. B. Same as above except cells were transfected with either a scrambled siRNA control or siCTCF before transfection of XhoI luciferase reporter constructs. **represents p<0.01. C. Same as panel B except cells were transfected with PstI luciferase reporter constructs. **represents p<0.01, *represents p<0.05.

We were intrigued by CTCF binding at *NANOG*-*DPPA3*-*GDF3* locus because CTCF flanks this 160 kb-large chromatin domain. CTCF has been implicated in global genome organization by regulating enhancer-blocking or barrier insulators [6]. We therefore explored whether CTCF-bound sites at this locus could function as enhancer-blocking insulators using a well characterized heterologous assay [34]. Our results revealed that all four CTCF-bound sites at the *NANOG* locus can function individually as enhancer blockers because they strongly decrease luciferase reporter activity when placed between the enhancer and the promoter (XhoI site) but not when placed upstream of the enhancer (PstI site) (Figure 4A). Interestingly, the degree of enhancer-blocking varied from site-to-site: the two CTCF-bound sites that flank the ∼160 kb locus, −151 and +17, show stronger enhancer-blocking activity than the internal –146 and –112 sites, which border the *APOBEC1* gene (Figure 3B). The activities of CTCF sites at –151 and +17 are also comparable to the 5′HS4 region from the chicken β-globin locus that has been shown previously to function as a potent enhancer-blocking insulator [11].

Next, we asked if the enhancer-blocking activity is CTCF-dependent. To test this, we ablated CTCF using siRNA before performing the heterologous enhancer-blocking assay. Upon CTCF depletion, the enhancer-blocking activity was abrogated by two-to three-fold (Figure 4B). Importantly, no significant effect was seen upon CTCF knockdown on sites cloned upstream of the enhancer (Figure 4C). However, the 5′HS4 site that was used as a positive control exhibited a weak effect when cloned upstream of the enhancer possibly reflecting a weak barrier activity on a transiently expressed template. Nonetheless, these data clearly demonstrate that CTCF recognition sequences within the *NANOG* locus can function as enhancer-blocking insulators in a CTCF-dependent manner, the strongest of these being the outermost sites at –151 and +17 kb that flank the entire ∼160 kb domain.

### CTCF Binding to the *NANOG* Locus is Invariant Upon Differentiation but CTCF Cofactors and Protein PARlation at Specific CTCF Sites are Selectively Modulated

**Figure 5:**
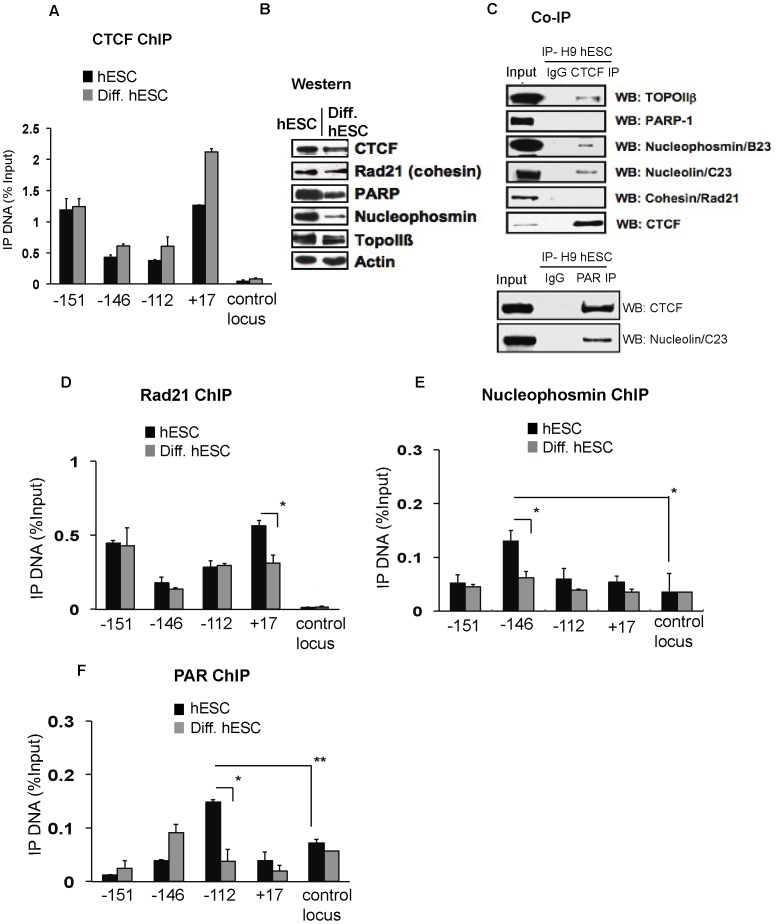
Differential association of CTCF interacting partners and protein poly(ADP- ribosyl)ation within the *NANOG* locus in pluripotent and differentiated hESCs. A. CTCF ChIP as in Figure 3 in H9 hESCs (black bars) and H9 hESCs differentiated with BMP4 for 5 days (Diff. hESC). The α-IgG (control) immunoprecipitates significantly lower levels of DNA relative to input (data not shown). Mean ± SEM represented. B. Western analyses of indicated proteins in H9 hESCs and 5 day BMP4-treated H9 hESCs (Diff. hESC). Mean ± SEM represented. C. Top panel: Immunoprecipitation of CTCF followed by Western blot analysis of indicated proteins. Bottom panel: Immunoprecipitation using an anti-PAR antibody followed by Western blot analysis with CTCF or Nucleolin. 1% Input was used in both top and bottom panels. D–F. As in Figure 3, except ChIP analysis of indicated proteins or modification. *represents p<0.05, **represents p<0.01. Mean ± SEM represented.

To examine the status of CTCF binding within the *NANOG* locus upon differentiation, we performed CTCF ChIP experiments in BMP4-treated hESCs. Interestingly, CTCF association does not significantly change even when the *NANOG* locus becomes transcriptionally repressed upon differentiation (Figures 5A and 1B). In fact, our results are consistent with genome-wide studies conducted across five different human cell lines as well as mouse ES cells. These studies showed that CTCF binding remains largely unaltered not only between cell lines but also across species [18], [19]. Importantly, our observation, in conjunction with other studies, raises an important question: although gene expression is highly cell type-specific, why is it that CTCF binding remains largely unchanged between cell types?

In this regard, it is interesting that CTCF can interact and/or co-localize with a variety of proteins, including Poly(ADP-ribosyl) Polymerase 1 (PARP1), TopoIIβ, Cohesin, Nucleophosmin and Nucleolin. Importantly, co-localization of some of these partners and post-translational modification by Poly(ADP-ribosyl)ation (PARlation) have been shown to be critical for CTCF-mediated insulator activity [17], [35]–[39]. Having established that CTCF binding sites within the *NANOG* locus can function as enhancer-blocking insulators, we next asked if known CTCF interacting partners co-localize at these CTCF sites. First, whole cell extracts from pluripotent ESCs showed high protein levels of CTCF, Cohesin, PARP1, Nucleophosmin, Nucleolin and TopoIIβ (Figures 5B). Second, when CTCF was immunoprecipitated from hESCs we detected the presence of TopoIIβ, Nucleophosmin and Nucleolin in multi-subunit complexes (Figure 5C). Third, we found that both CTCF and Nucleolin are poly(ADP-ribosyl)lated (PARlated) in pluripotent hESCs (Figure 5C). These observations are consistent with our findings in human breast cancer cells that multi-subunit complexes containing PARlated CTCF and Nucleolin are required to maintain a stable chromatin boundary upstream of the tumor suppressor *p16* gene, which protects it from aberrant epigenetic silencing [17]. Incidentally, CTCF-Cohesin localization has been shown to be important for insulator function and direct interaction between Cohesin and CTCF was recently demonstrated [40].

Having confirmed that some of the known CTCF binding partners interact with CTCF in hESCs, we next analyzed the localization of these partners with CTCF-bound insulator sites within the *NANOG* locus. In pluripotent hESCs, these sites were associated strongly with Rad21 (a subunit of Cohesin) (Figures 5D, black bars). Interestingly, Nucleophosmin was significantly enriched only at the −146 CTCF site (Figure 5E, black bars). Additionally, we tested for the presence of chromatin-associated PARlated proteins in hESCs and found significant enrichment only at −112 (Figure 5F, black bars) From these data, it is clear that distinct protein partners and protein PARlation are present at different CTCF-bound insulator elements.

Next, we investigated the changes in *NANOG*-bound CTCF complexes that occur during differentiation. We noticed a decrease in cellular protein levels of CTCF, PARP1 and Nucleophosmin upon BMP4-induced hESC differentiation, whereas Rad21 (Cohesin) and TopoIIβ levels did not change (Figure 5B). Interestingly, upon differentiation CTCF cofactors and protein PARlation were selectively lost from CTCF-associated sites: Rad21 binding decreased from the +17 site but remained unaltered at other sites (Figure 5D, gray bars) and Nucleophosmin was significantly weakened at −146 (Figure 5E, gray bars). Furthermore, we observed decreased protein PARlation at the −112 site (Figure 5F, gray bars). Based on these observations, we suggest that although CTCF binding is relatively unchanged between pluripotent and differentiated hESCs, functionally and compositionally distinct CTCF complexes may associate with different sites within the *NANOG* locus.

### Different CTCF Cofactors are Present at Distinct CTCF Sites on Critical Pluripotency Genes

Next, we analyzed CTCF association with other important stem cell genes. *cMYC* is a known CTCF-regulated gene and CTCF has been shown to associate with both the distal and proximal promoters of *cMYC* [41], [42]. As expected, CTCF binds to both promoter sites in hESCs albeit more strongly at the distal promoter (Figure S7A). In addition, CTCF associates 2.2 kb upstream of *SOX2* and in an *LIN28* intron (Figure S7A).

We then asked if distinct CTCF complexes are present at other CTCF-regulated stem cell genes. Interestingly, Rad21 strongly associates with CTCF sites at the *cMYC* and *SOX2* genes but weakly with *LIN28* (Figure S7B). We observed very weak enrichment of Nucleophosmin at *cMYC* and *LIN28* (Figure S7C). These data again substantiate the notion that distinct CTCF complexes associate with stem cell genes.

In summary, we show that CTCF interacts with several gene loci that are critical for self-renewal and proliferation of hESCs. Upon CTCF depletion, expression of certain pluripotency genes decreases and this correlates with decreased CTCF binding from particular site(s): –146 and –112 within the *NANOG-DPPA3-GDF3* locus and the *cMYC* proximal site (Figures 2D, S8 and S9). In addition, the proliferation defect in CTCF-depleted cells could be attributed to direct regulation of *cMYC* by CTCF through association with the distal and proximal promoters. Importantly, the position where CTCF binds to a given gene locus varies from one gene to the other, highlighting the distinct mechanisms by which CTCF could possibly regulate stem cell genes as either an activator, repressor or insulator.

## Discussion

CTCF is a ubiquitous protein that can regulate chromatin “barrier” boundaries or enhancer-blocking insulators in addition to functioning as a transcriptional activator or repressor (reviewed in Filippova, 2008). Several studies have shown that CTCF plays important roles in processes such as genetic imprinting, X-chromosome inactivation and preventing aberrant transcriptional silencing of tumor suppressor genes [6], [17]. Here we show that CTCF depletion significantly accelerates BMP4-induced differentiation and to a lesser extent TPA- and retinoic acid-induced differentiation pathways without inducing spontaneous differentiation. In addition, we find that CTCF depletion affects hESC proliferation and expression of genes that regulate pluripotency. Consistent with our results, CTCF has been shown to positively influence cell cycle progression of αβ T cells in that CTCF depletion results in increased expression of the cell-cycle arrest gene, *p21*, concomitant with decreased proliferation [43]. Furthermore, our data demonstrate that CTCF physically associates with pluripotency gene loci and potentially insulates some target genes from the effects of transcriptional repression. *NANOG*, *OCT4* and *SOX2* are essential genes for ESC pluripotency; *NANOG* participates in a positive regulatory feedback loop with *OCT4* and *SOX2* and plays a key role in activating other genes that are components of critical stem cell signaling pathways [3]. We show that four CTCF binding sites flank the *NANOG-DPPA3-GDF3* locus at −151, −146, −112, and +17 kb and that CTCF depletion negatively impacts *NANOG* and *DPPA3* expression in hESCs (p<0.05 respectively at 96 hrs for both). Interestingly, mRNA levels of *GDF3* increase upon CTCF knockdown possibly because this gene is transcribed in the opposite direction from *NANOG* and *DPPA3* or a previously restricted enhancer within the locus has become available to the *GDF3* promoter.

We further establish that the four CTCF binding sites within the *NANOG* locus can each function as an enhancer-blocking insulator in a heterologous system. In particular, the sites flanking the *NANOG* locus at –151 and +17 kb have the highest affinity for CTCF, since these sites remain associated even upon siRNA-mediated CTCF ablation, and are the most effective as enhancer-blocking insulators. Thus, we hypothesize that the outermost CTCF recognition sites at –151 and +17 kb may segregate the multi-gene *NANOG* locus into a discrete chromosomal domain and shield it from the effect of surrounding regions by serving as barrier insulators.

The human chr12p13 is a hotspot for teratocarcinomas and synchronized over-expression of the *NANOG-DPPA3-GDF3* genes has been reported in human embryonal carcinomas, seminomas and breast carcinomas [44], [45]. Notably, this gene cluster is also highly transcribed in hESCs and the coordinated expression of these genes decreases upon BMP4-induced differentiation and formation of embryoid bodies (this study and [46]). We speculate that this region may be organized into a higher-order chromatin structure by possible intra-chromosomal looping and that this association changes upon differentiation. In fact, a similar looping phenomenon was postulated to occur between the two distal hypersensitive sites at the *β-globin* locus [47]. Studies in mouse ESCs identified several CTCF-associated sites approximately 50 kb upstream of the *NANOG* transcription start site [28] [48]. Using a CTCF consensus prediction program (http://insulatordb.uthsc.edu, [32]), we identified CTCF consensus sites at −37 kb and −22 kb upstream of the human *NANOG* transcription start site; however, we did not detect CTCF enrichment by ChIP at these sites in hESCs (data not shown). Consistent with our data, global ChIP-seq analysis in hESCs did not reveal CTCF interaction in the vicinity around ∼50 kb upstream of the human *NANOG* locus [18]. This suggests species-specific regulation of the *NANOG* gene locus or possible differences within the murine and human ES cell regulatory network.

In addition to the *NANOG l*ocus, we found that CTCF also associates with *SOX2*, *cMYC* and *LIN28* loci. Interestingly, CTCF interaction with these genes varies in location: CTCF flanks the *NANOG-DPPA3-GDF3* locus; CTCF binds to distal promoters of *cMYC* and *SOX2*, to proximal promoter of *cMYC*; and finally, CTCF associates within gene coding region of *LIN28*. This binding diversity may indicate that CTCF controls these genes by multiple mechanisms. We show that the four CTCF sites within the *NANOG* locus can function as enhancer-blocking insulators in a heterologous system. It is plausible that CTCF is an activator of *cMYC* transcription by associating with their proximal promoters. By contrast, at the *cMYC* and *SOX2* distal promoters, CTCF may provide a boundary or insulator activity. Importantly, CTCF was originally characterized as a repressor of *cMYC* transcription. However, a recent study demonstrated that at the endogenous *cMYC* site, CTCF is required for *cMYC* transcription and that deletion of distal and proximal CTCF sites at the native locus results in progressive encroachment of DNA methylation [41], [49]. Interestingly, CTCF binds to an intragenic site within *LIN28* and is important for maintaining its proper expression since CTCF depletion reduces *LIN28* mRNA levels. In fact, a large number of CTCF sites are intragenic and it has been suggested that these sites can regulate transcription by modulating RNA polymerase II progression [6], [33].

Consistent with published studies, which show that CTCF-genomic interaction remains largely indistinct between different cell types or species [18], [19], we found that CTCF binding within the *NANOG* locus is largely unaltered upon BMP4-induced differentiation or in terminally differentiated fibroblasts (Figure 5A and data not shown). Therefore, the genome appears to be punctuated by stable, invariant CTCF binding even when the transcriptional capacity, epigenetic patterns, and nuclear organization are quite dynamic. Interestingly, we observed site-selective changes in Nucleophosmin, Cohesin and protein PARlation near specific CTCF binding sites within the *NANOG* locus upon BMP4-induced differentiation. Thus CTCF cofactors could be selectively exchanged or post-translationally modified during cell fate commitment to modulate CTCF activity. Furthermore, our data indicates that both CTCF and Nucleolin are PARlated in hESCs and PARlated CTCF displays different affinities for its interaction partners, consistent with previous reports in human breast cancer cell lines [17].

Upon siRNA-mediated CTCF depletion, CTCF association is lost from two internal sites within the *NANOG* locus, −146 and −112, but not from the 5′ and 3′ flanking sites at −151 and +17, which also exhibit the strongest insulator activities in our heterologous assays (Figure 4). In general, we find that CTCF remains stably bound to a subset of other high-affinity sites even after efficient depletion of cellular CTCF protein (Figures S8A and S9). A possible explanation for this phenomenon is that the high-affinity sites have critical roles in cell viability, perhaps through maintaining higher-order chromosomal structures. By contrast, the lower-affinity sites may regulate more restricted, gene-specific activities, which are dispensable for cell survival. Thus, upon transient CTCF knockdown, 20% of the remaining CTCF molecules may remain bound to such high-affinity sites to prevent catastrophic loss of cell integrity.

CTCF is known to mediate intra- as well as inter-chromosomal interactions [6], [14], [47] and can tether target genes to the nucleolus through association with Nucleolin or Nucleophosmin, as described for the chicken 5′-HS4 insulator transgene in K562 cells [35]. In hESCs, CTCF could employ similar mechanisms to compartmentalize the genome to form a “stem cell transcription hub”. Upon cell fate commitment, such CTCF-dependent tethering may be reconfigured by modulation of contact between CTCF molecules or dissociation of cofactors, such as Cohesin or nucleolar protein(s), from CTCF complexes. Further studies that elucidate the function of CTCF-cofactor interactions at specific genomic locations will be valuable towards understanding how pluripotency is maintained in human ES cells and lost upon differentiation.

## Materials and Methods

### hESC Culture

Human Embryonic Stem Cell (hESC) lines H9 (WA09) or H1 (WA01) were grown on Matrigel (BD Bioscience) in mTeSR1 (Stem Cell Technologies) or an in-house version (Salk Stem Cell Core). For differentiation, 200 ng/ml recombinant BMP4 (Stemgent) was added to TeSR for five days unless otherwise mentioned. Media was replenished every 24 hrs. For TPA-induced differentiation, 50 ng/ml TPA was added directly to TeSR. For retinoic acid induced differentiation, hESCs were grown in TeSR media minus FGF and TGFβ growth factors prior to differentiation using 1 µM retinoic acid. For Embryoid Body (EB) differentiation, cells were displaced by dispase treatment and re-suspended in EB media (DMEM/F12 containing 20% FBS, 1% NEAA and 1% glutamax). Media was replenished every 48–72 hrs.

### Chromatin Immunoprecipitation (ChIP)

hESCs, cultured in 10cm dishes, were cross-linked for 15 minutes by addition of formaldehyde (1% final concentration) to TeSR. Cross-linking was stopped upon addition of Glycine to a final concentration of 125 mM for 5 minutes. After washing with 1X PBS thrice, cells were scraped and collected in RIPA (150 mM NaCl, 1% NP-40, 0.5% Sodium Deoxycholate, 0.1% SDS, 50 mM Tris-HCl pH 8.0, 5 mM EDTA) containing protease inhibitors. Lysates were subsequently sonicated and 500 µg of lysate was pre-cleared for 1 hour using 40 µl of a 50% slurry of 1∶1 protein A- and protein G-Sepharose beads (GE Healthcare). For immunoprecipitation, the indicated antibodies were added to pre-cleared lysates along with 40 µl of a 50% slurry of 1∶1 protein A/G beads and incubated at 4°C overnight. Antibodies used were: α-CTCF (Millipore), α-Rad21 (Abcam), α-Nucleophosmin (Santa Cruz), α-TopoIIβ (Santa Cruz) and α-PAR (Millipore). Catalog number and lot number information is included in Table S2. Immuno-complexes were washed twice with RIPA, thrice with IP wash buffer (100 mM Tris-HCl pH 8.5, 500 mM LiCl, 1% NP-40, 1% Sodium Deoxycholate) and twice with 1X TE before eluting in 200 µl of buffer containing 70 mM Tris-HCl pH 8.0, 1 mM EDTA and 1.5% SDS after incubation at 65°C for 10 or 30 minutes. Crosslinks were reversed from immuno-complexes by addition of 200 mM NaCl and incubation at 65°C for 6 hours or overnight. DNA was purified by incubation with proteinase K and phenol-chloroform extraction. Input samples were treated similarly and associated DNA was identified by qPCR. PCR primers are listed in Table S1. Statistical analyses were performed using unpaired two-tailed student’s *t-*test.

### CTCF Knockdown

For CTCF depletion, hESCs were collected as single cells using TrypLE (Gibco), washed and plated in TeSR containing ROCK inhibitor Y27632. The next day, cells were transfected with 100 nM scrambled siRNA (Ambion) or a pre-designed Silencer Select siRNA against CTCF (Ambion; siRNA IDs s20966, s20967, s20968) using lipofectamine RNAiMAX (Invitrogen) according to the manufacturer’s instructions. 24 hours later, cells were transfected again. In Figure 2D, 40,000 cells were plated in a 24-well dish whereas in Figures 2E and F, 20,000 cells were plated. For the immunofluorescence experiment in Figure S4, hESCs were transfected thrice with control or CTCF siRNA before fixation.

### RT-PCR

For gene expression analysis, cells were collected by trypsinization and RNA was extracted using RNAeasy (Qiagen) and subjected to DNAse (Invitrogen) treatment according to the manufacturer’s instructions. cDNA was synthesized from 0.5 to 1 µg of RNA using Superscript III Reverse transcriptase (Invitrogen) according to the manufacturer’s protocol and subjected to qPCR using the primers listed in Table S1.

### Western Blot Analysis and Co-immunoprecipitation

For immunoblotting, cells were lysed with RIPA containing protease inhibitors. 25 or 50 µg of protein was resolved on SDS gels, transferred to nitrocellulose membranes and proteins were recognized using the following antibodies: α-CTCF (BD Bioscience), α-Rad21 (Abcam), α-PARP (Santa Cruz), α-Nucleophosmin (Santa Cruz), α-TopoIIβ (Santa Cruz), α-Nanog (Abcam), α-Oct4 (Santa Cruz), α-Sox2 (Chemicon), α-Actin (Sigma). Catalog number and lot information are provided in Table S2.

For co-immunoprecipitation, cells were collected by trypsinization and lysed using twice the volume of whole cell extract lysis buffer (20 mM Tris HCl pH 7.5, 420 mM NaCl, 2 mM MgCl_2_, 1 mM EDTA, 10% glycerol, 0.5% NP-40, 0**.**5% Triton-X-100) for 30 to 45 minutes on ice. Supernatant was recovered after centrifugation, 2 mg lysate was diluted 6X with IP buffer (20 mM Tris HCl pH 7.5, 50 mM NaCl, 10 mM MgCl2, 2 mM EDTA, 0.5% Triton-X-100) and pre-cleared using 50 µl of protein-G Sepharose beads for 2 hours. To pre-cleared lysates, 2–5 µg of antibody was added and incubated overnight at 4°C. Subsequently, 50 µl of a 50% slurry of protein-G beads was added and incubated for 4 hours. Immunocomplexes were washed with IP buffer containing 0.5% Triton-X-100 thrice and once with IP buffer containing 0.1% Triton-X-100. 25 µl of 2X SDS loading buffer was added and boiled for 10 minutes prior to SDS-PAGE and Western blotting using the antibodies indicated above.

### Immunofluorescence

hESCs were grown and/or differentiated on coverslips pre-coated with Matrigel. Cells were washed, fixed with 4% paraformaldehyde for 10 minutes and permeabilized using 0.4% Triton-X-100. Permeabilized cells were blocked with 5% FBS for 30 minutes and incubated with α-Nanog (Abcam) or α-Oct4 (Santa Cruz) antibodies overnight. Coverslips were washed with 1% BSA solution and incubated with secondary antibodies conjugated with FITC or Alexa568 fluorophores. DAPI (Sigma) staining was performed along with secondary antibody incubation. Coverslips were mounted on a glass slide using Vectashield (Vector laboratories) and visualized on a Leica SP2 confocal microscope. Image contrasts and brightness parameters were adjusted across all treatment groups where necessary. Images were acquired in a sequential mode of the fluorescent channels to prevent fluorescence bleed-through.

### Enhancer-blocking Assay

Enhancer-blocking assays were performed as described in Lunyak et al [50]. Enhancer-blocking plasmids contained a CMV enhancer and CMV promoter-driven luciferase reporter and 1.2 kb of DNA encompassing CTCF binding sites from relevant regions were cloned either in XhoI or Pst1 restriction enzyme sites. Briefly, 293T cells were grown in DMEM (high glucose) and 10% FBS. 1.5×10^5^ cells were plated in 24-well dishes and transfected with 100 ng of plasmid when cells were 70% confluent (approximately 24 hours later) using lipofectamine 2000 (Invitrogen). Plasmids were linearized using an enzyme downstream of the luciferase reporter (KpnI or Apa1). 24 hours after transfection, luciferase activity was measured using the Dual-luciferase reporter assay system (Promega). Luciferase activity was normalized to a co-transfected renilla-luciferase reporter plasmid and represented relative to the luciferase/renilla activity of an empty pELuc plasmid set to 100%. Statistical analyses were performed using unpaired two-tailed student’s *t-*test.

In Figures 4B and C, cells were transfected with 100 nM scrambled siRNA (Ambion) or a pre-designed Silencer Select siRNA against CTCF (Ambion) using lipofectamine RNAiMAX (Invitrogen) according to the manufacturer’s instructions. 24 hours later, cells were transfected again with siRNAs. 48 hours after the first siRNA transfection, plasmids were transfected as described above.

### Proliferation Assay

2500 cells were plated in a 96-well dish and siRNA transfections were performed as described above. Proliferation was measured using a BrdU cell proliferation assay kit (Chemicon International) according to the manufacturer’s protocol. Briefly, approximately 48 hours after the first transfection, BrDU was pulsed overnight. Subsequently, cells were fixed and BrdU was detected using a α-BrdU antibody followed by a peroxidase-conjugated secondary antibody. Absorbance at 450 nm was measured on a plate reader upon addition of a substrate for peroxidase. BrdU incorporation in CTCF-depleted cells was normalized to that of control-transfected cells set to 1.

For cell death assays, PI was added to trypsinized cells and extent of PI staining was measured on BD FACSCalibur flow cytometer.

### hCG ELISA

Spent media were collected from hESCs before and after differentiation. One-fifth of the spent media was used for each ELISA. hCG ELISA was performed using the hCG ELISA kit (Calbiotech) as per manufacturer’s instructions.

## Supporting Information

Figure S1 **Morphology of hESCs after CTCF knockdown.** A. Western analyses showing kinetics of CTCF knockdown in H9 hESCs. Control si represents a scrambled siRNA control. Time points represent time after first siRNA transfection. For details regarding the experimental design, see Figure 2C. B. Phase contrast images of H9 hESCs transfected with a control si or siCTCF at 4X and 10X magnifications.
(TIF)

Figure S2 **Confirmation of CTCF depletion phenotype using additional siRNAs.** A. Western analysis of CTCF upon CTCF depletion using additional siRNAs (2) and (3) in comparison to a scrambled control (control si) in H9 hESCs. B. Phase contrast images of H9 hESCs at 4X magnification transfected with indicated siRNAs. C. mRNA levels of indicated genes at 48 hrs after CTCF knockdown in H9 hESCs. mRNA levels of indicated genes in control si and siCTCF were normalized to respective *GAPDH* levels. Subsequently, mRNA levels of siCTCF were normalized to control si set to 1. siRNA (1) represents siRNA used in Figure 2; siRNA (2) was used for confirmation.
(TIF)

Figure S3 **Cell death analysis of control si and siCTCF transfected cells.** 72 hrs after siRNA transfection, attached cells and cells in supernatant were collected and pooled. Collected cells were stained with propidium iodide and analyzed by BD FACSCalibur. Following flow cytometry, cells were collected back, lysed and analyzed by western blot analysis. For a positive control, untransfected H9 hESCs were treated with 1 mM hydrogen peroxide for 4 hrs (bottom left panel).
(TIF)

Figure S4 **Representative images of immunofluorescence analysis of NANOG and OCT4 proteins 96 hrs after CTCF depletion.** Scale bar represents 150 µm.
(TIF)

Figure S5 **Loss of pluripotency upon CTCF depletion depends on the differentiation protocol.** RT-PCR analysis of indicated genes upon control si (black line) or siCTCF (red line) transfection followed by TPA treatment (50 ng/ml) for 2 days in H9. mRNA levels were analyzed at the indicated days and normalized to *GAPDH* and further normalized to vehicle (DMSO) control. **represents p<0.01, *represents p<0.05. A. RT-PCR analysis of indicated genes upon control si or siCTCF transfection followed by 1 µM Retinoic Acid treatment for 48 hrs in H9. mRNA levels are normalized to *GAPDH* and further normalized to vehicle (DMSO) control. *represents p<0.05. B. RT-PCR analysis of indicated genes upon control si or siCTCF transfection followed by embryoid body formation. mRNA levels of represented genes in both groups was analyzed on day 10 after embryoid body formation, normalized to *GAPDH* and further normalized to control si set to 1.
(TIF)

Figure S6 **Consensus motifs of CTCF recognition sites in the** ***NANOG*** **locus.** Top: CTCF consensus motif as identified in [33]. CTCF consensus motifs at the *NANOG* locus were identified using a prediction algorithm as described in [32] and http://insulatordb.uthsc.edu/. Asterisks indicate a perfect match of the denoted base to the consensus. Hyphen indicates lack of a match. A. Browser snapshot of CTCF ChIP-seq data from the *NANOG-DPPA3-GDF3* locus in H1 hESCs from the UCSC genome browser. Asterisks represent the sites that we have identified and characterized in H9 hESCs in this study.
(TIF)

Figure S7 **Different CTCF cofactors are present at distinct CTCF sites on several critical pluripotent genes.** A. ChIP analyses as in Figure 3B except at indicated genes in H9 hESCs. Key: Prom  =  promoter, prox  =  proximal, int  =  intron and ctrl  =  a control region for ChIP near the pertinent gene of interest. Position of the amplicon is either as indicated in the figure or text, relative to the transcription start site of the respective gene. Mean ± SEM represented. B and C. ChIP analysis as in panel A, except for Cohesin (Rad21) and Nucleophosmin (B23). Mean ± SEM represented. For all panels, statistical significance has been calculated for enrichment over the respective negative control region. *represents p<0.05, **represents p<0.01.
(TIF)

Figure S8 **ChIP analyses of protein binding to the** ***NANOG*** **locus in H9 hESCs when transfected with control si or siCTCF.** Statistical significance in panel C depicts fold enrichment of nucleophosmin at –146 over the negative control region.
(TIF)

Figure S9 **ChIP analyses of CTCF binding at indicated loci in H9 hESCs when transfected with control si or siCTCF.**
(TIF)

Table S1 **Primer sequences for ChIP-qPCR and RT-qpCR experiments.**
(XLSX)

Table S2 **Antibodies used in this study, along with vendor name, catalog number and lot information (where available).**
(XLSX)
