# Supplementary figures and images for: Functional and Molecular Characterization of the Role of CTCF in Human Embryonic Stem Cell Biology
Source: PLoS One. 2012 Aug 3;7(8):e42424. doi: 10.1371/journal.pone.0042424 (PMC3411781; doi:10.1371/journal.pone.0042424)

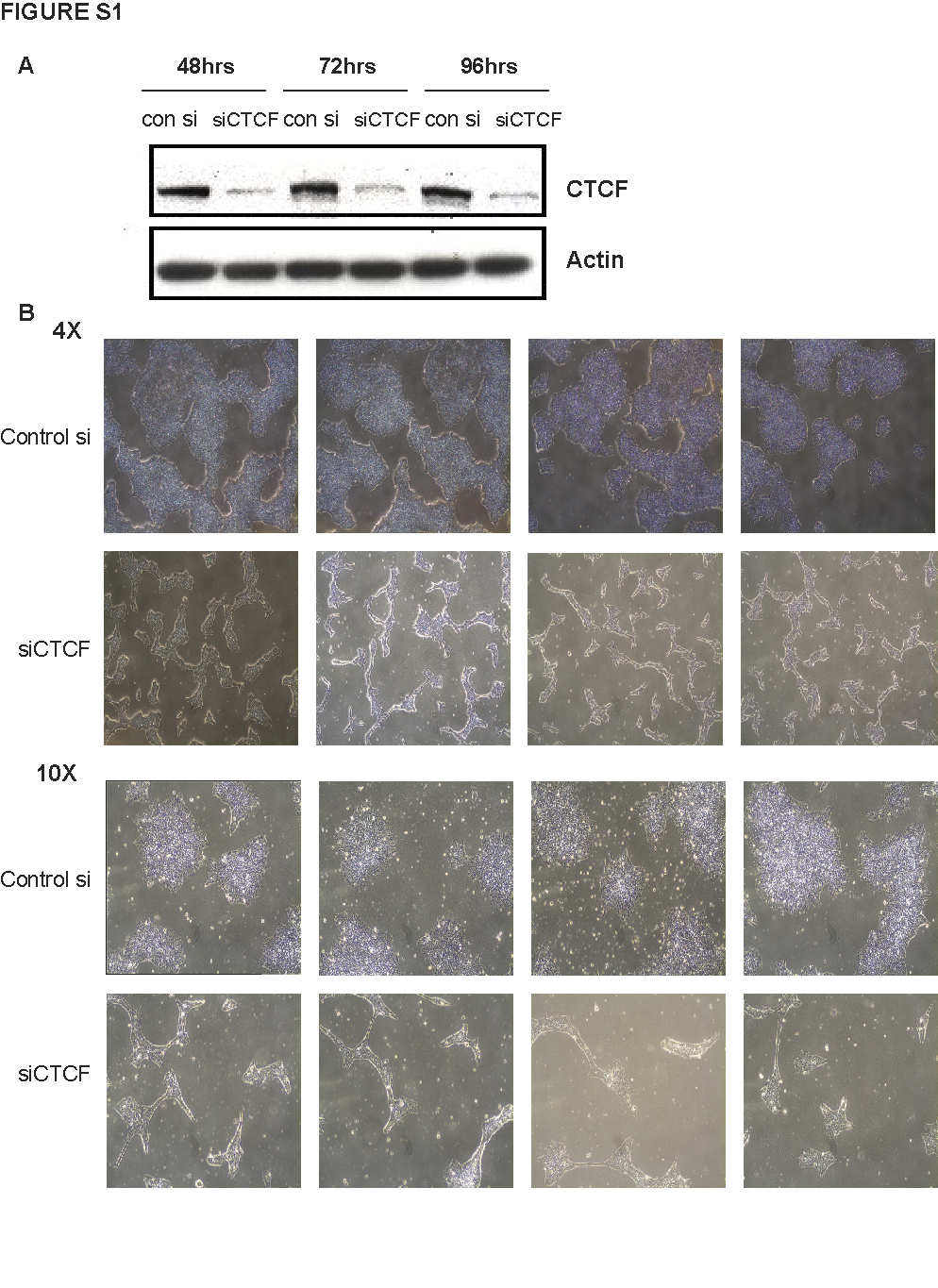

Supplement: Figure S1 — Morphology of hESCs after CTCF knockdown. A. Western analyses showing kinetics of CTCF knockdown in H9 hESCs. Control si represents a scrambled siRNA control. Time points represent time after first siRNA transfection. For details regarding the experimental design, see Figure 2C. B. Phase contrast images of H9 hESCs transfected with a control si or siCTCF at 4X and 10X magnifications. (TIF) [file pone.0042424.s001.tif]

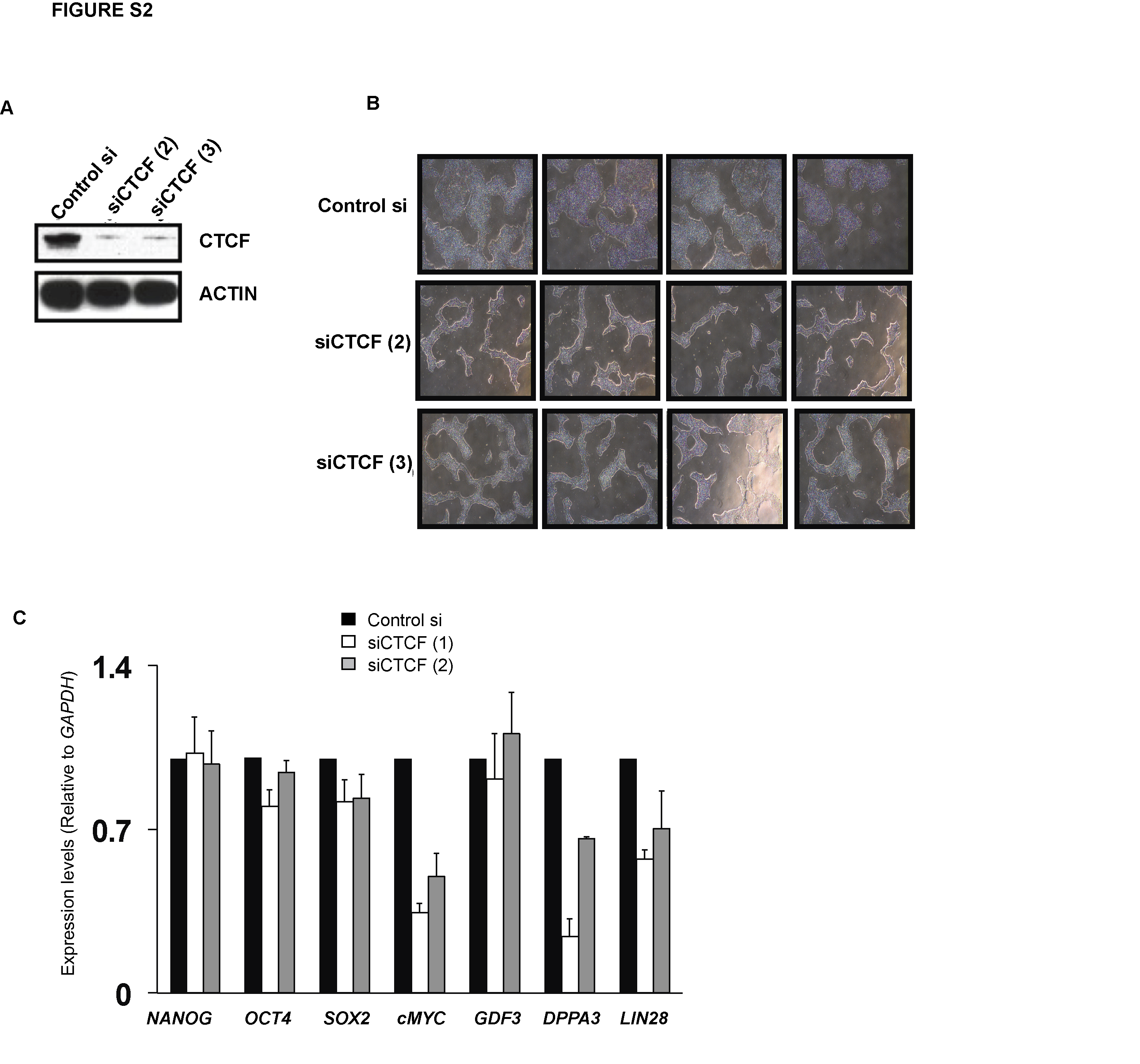

Supplement: Figure S2 — Confirmation of CTCF depletion phenotype using additional siRNAs. A. Western analysis of CTCF upon CTCF depletion using additional siRNAs (2) and (3) in comparison to a scrambled control (control si) in H9 hESCs. B. Phase contrast images of H9 hESCs at 4X magnification transfected with indicated siRNAs. C. mRNA levels of indicated genes at 48 hrs after CTCF knockdown in H9 hESCs. mRNA levels of indicated genes in control si and siCTCF were normalized to respective GAPDH levels. Subsequently, mRNA levels of siCTCF were normalized to control si set to 1. siRNA (1) represents siRNA used in Figure 2; siRNA (2) was used for confirmation. (TIF) [file pone.0042424.s002.tif]

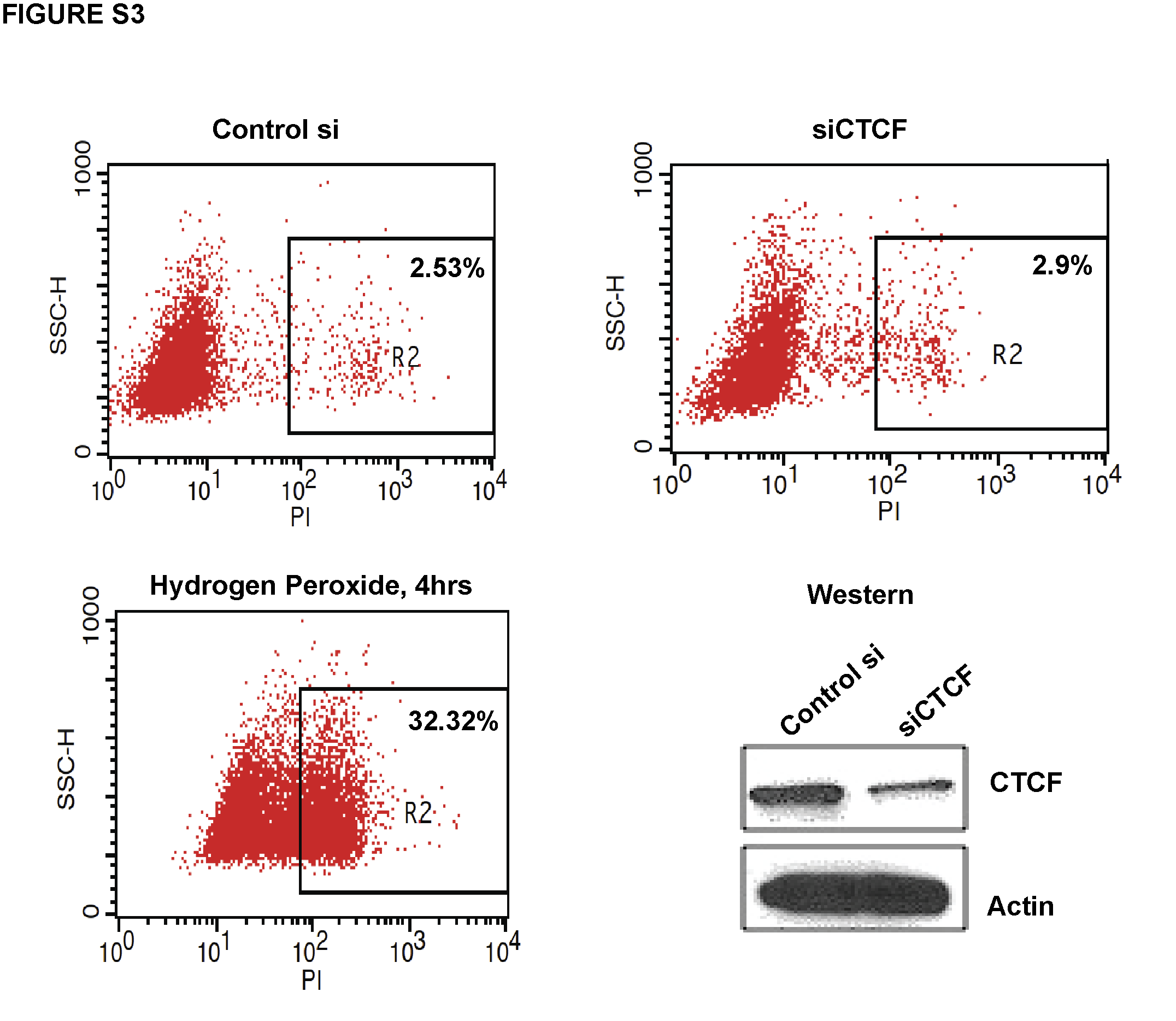

Supplement: Figure S3 — Cell death analysis of control si and siCTCF transfected cells. 72 hrs after siRNA transfection, attached cells and cells in supernatant were collected and pooled. Collected cells were stained with propidium iodide and analyzed by BD FACSCalibur. Following flow cytometry, cells were collected back, lysed and analyzed by western blot analysis. For a positive control, untransfected H9 hESCs were treated with 1 mM hydrogen peroxide for 4 hrs (bottom left panel). (TIF) [file pone.0042424.s003.tif]

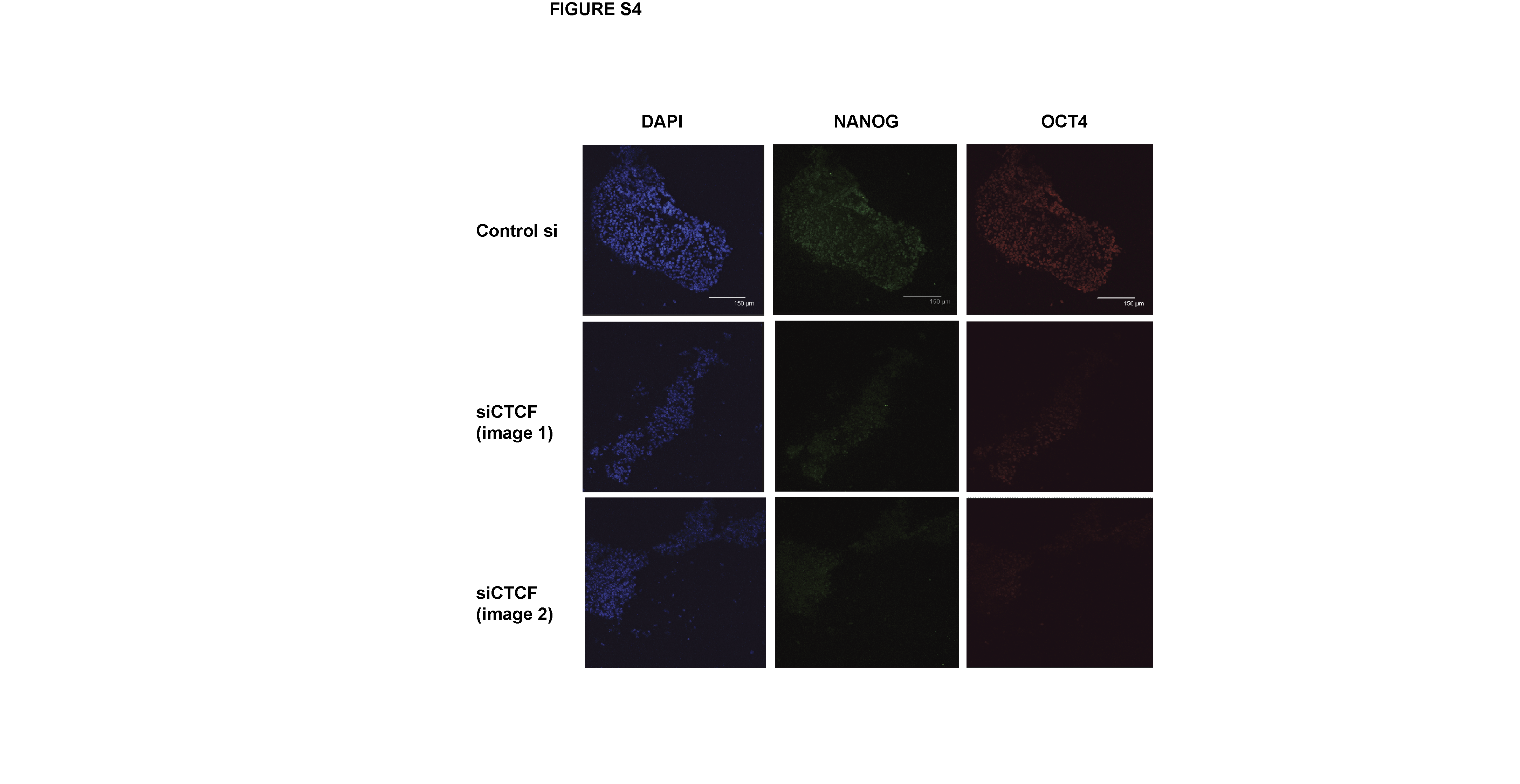

Supplement: Figure S4 — Representative images of immunofluorescence analysis of NANOG and OCT4 proteins 96 hrs after CTCF depletion. Scale bar represents 150 µm. (TIF) [file pone.0042424.s004.tif]

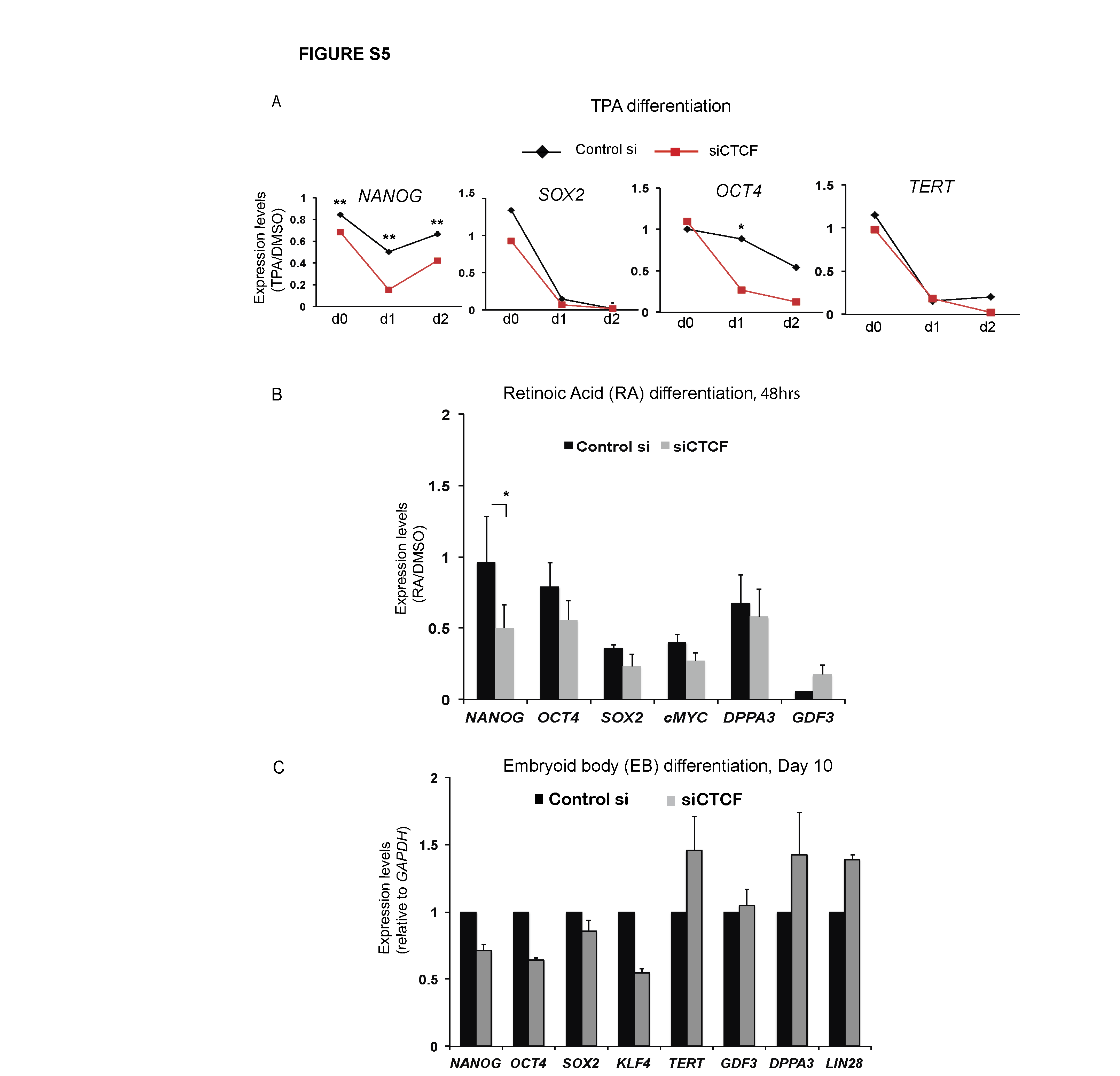

Supplement: Figure S5 — Loss of pluripotency upon CTCF depletion depends on the differentiation protocol. RT-PCR analysis of indicated genes upon control si (black line) or siCTCF (red line) transfection followed by TPA treatment (50 ng/ml) for 2 days in H9. mRNA levels were analyzed at the indicated days and normalized to GAPDH and further normalized to vehicle (DMSO) control. **represents p<0.01, *represents p<0.05. A. RT-PCR analysis of indicated genes upon control si or siCTCF transfection followed by 1 µM Retinoic Acid treatment for 48 hrs in H9. mRNA levels are normalized to GAPDH and further normalized to vehicle (DMSO) control. *represents p<0.05. B. RT-PCR analysis of indicated genes upon control si or siCTCF transfection followed by embryoid body formation. mRNA levels of represented genes in both groups was analyzed on day 10 after embryoid body formation, normalized to GAPDH and further normalized to control si set to 1. (TIF) [file pone.0042424.s005.tif]

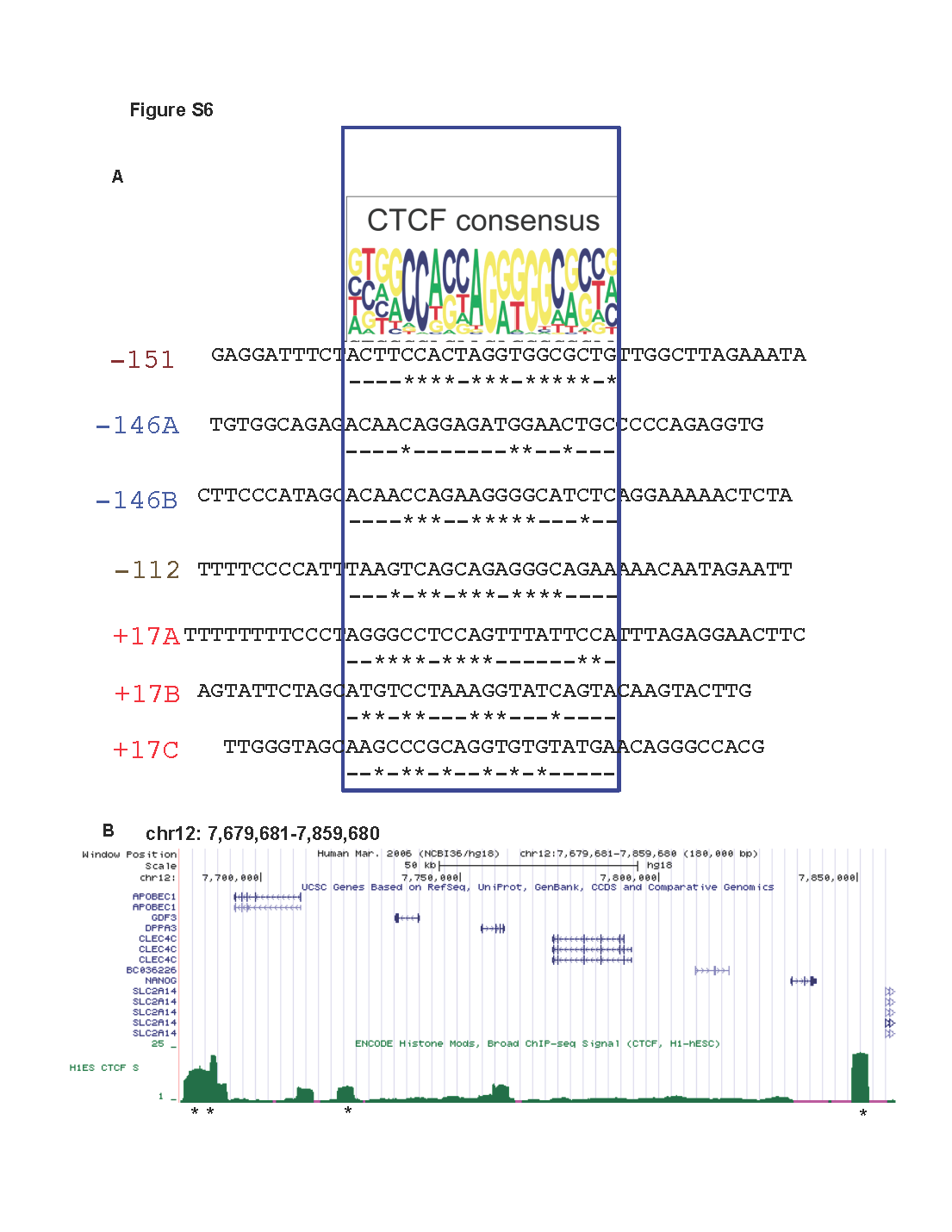

Supplement: Figure S6 — Consensus motifs of CTCF recognition sites in the NANOG locus. Top: CTCF consensus motif as identified in [33]. CTCF consensus motifs at the NANOG locus were identified using a prediction algorithm as described in [32] and http://insulatordb.uthsc.edu/. Asterisks indicate a perfect match of the denoted base to the consensus. Hyphen indicates lack of a match. A. Browser snapshot of CTCF ChIP-seq data from the NANOG-DPPA3-GDF3 locus in H1 hESCs from the UCSC genome browser. Asterisks represent the sites that we have identified and characterized in H9 hESCs in this study. (TIF) [file pone.0042424.s006.tif]

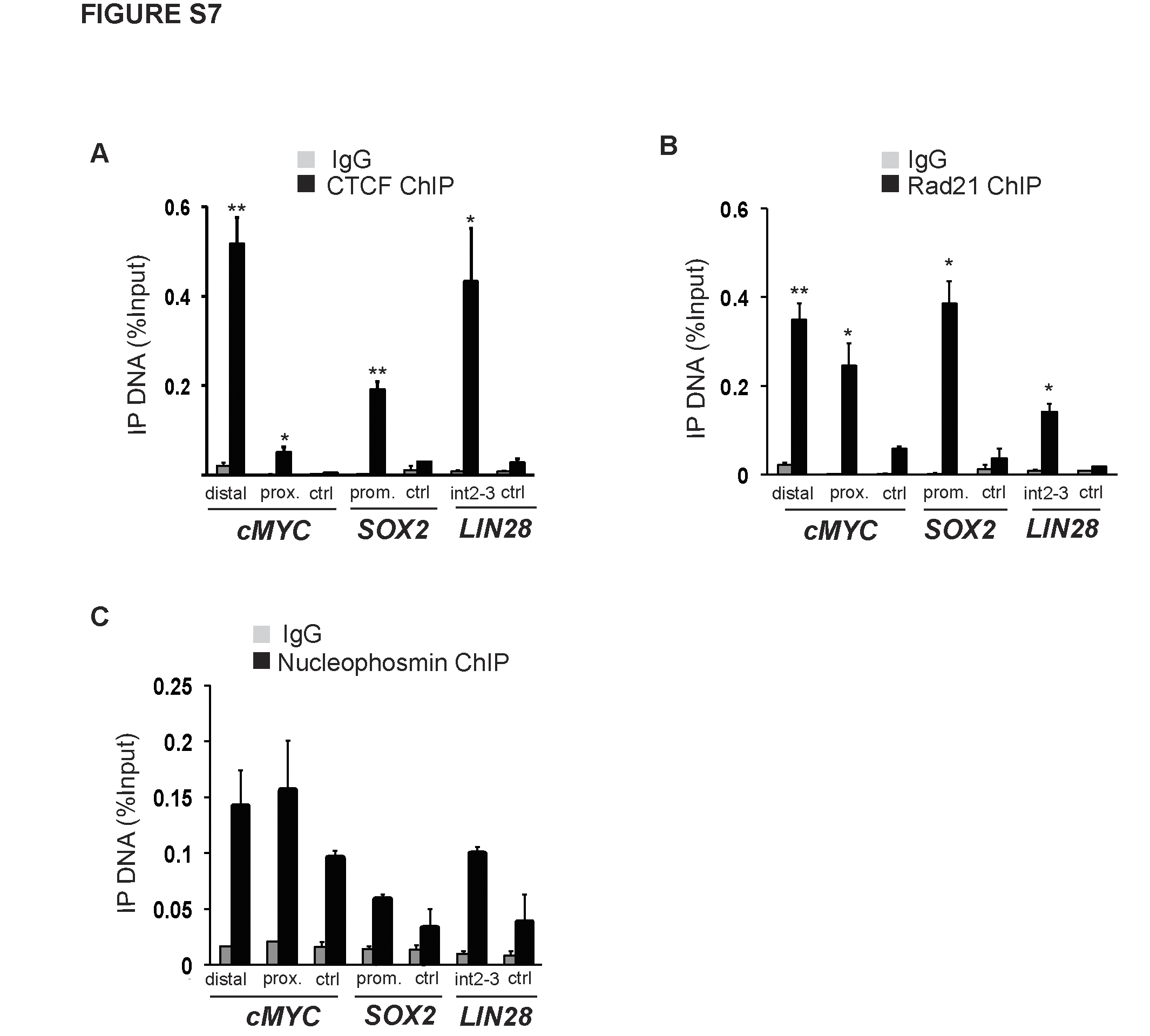

Supplement: Figure S7 — Different CTCF cofactors are present at distinct CTCF sites on several critical pluripotent genes. A. ChIP analyses as in Figure 3B except at indicated genes in H9 hESCs. Key: Prom = promoter, prox = proximal, int = intron and ctrl = a control region for ChIP near the pertinent gene of interest. Position of the amplicon is either as indicated in the figure or text, relative to the transcription start site of the respective gene. Mean ± SEM represented. B and C. ChIP analysis as in panel A, except for Cohesin (Rad21) and Nucleophosmin (B23). Mean ± SEM represented. For all panels, statistical significance has been calculated for enrichment over the respective negative control region. *represents p<0.05, **represents p<0.01. (TIF) [file pone.0042424.s007.tif]

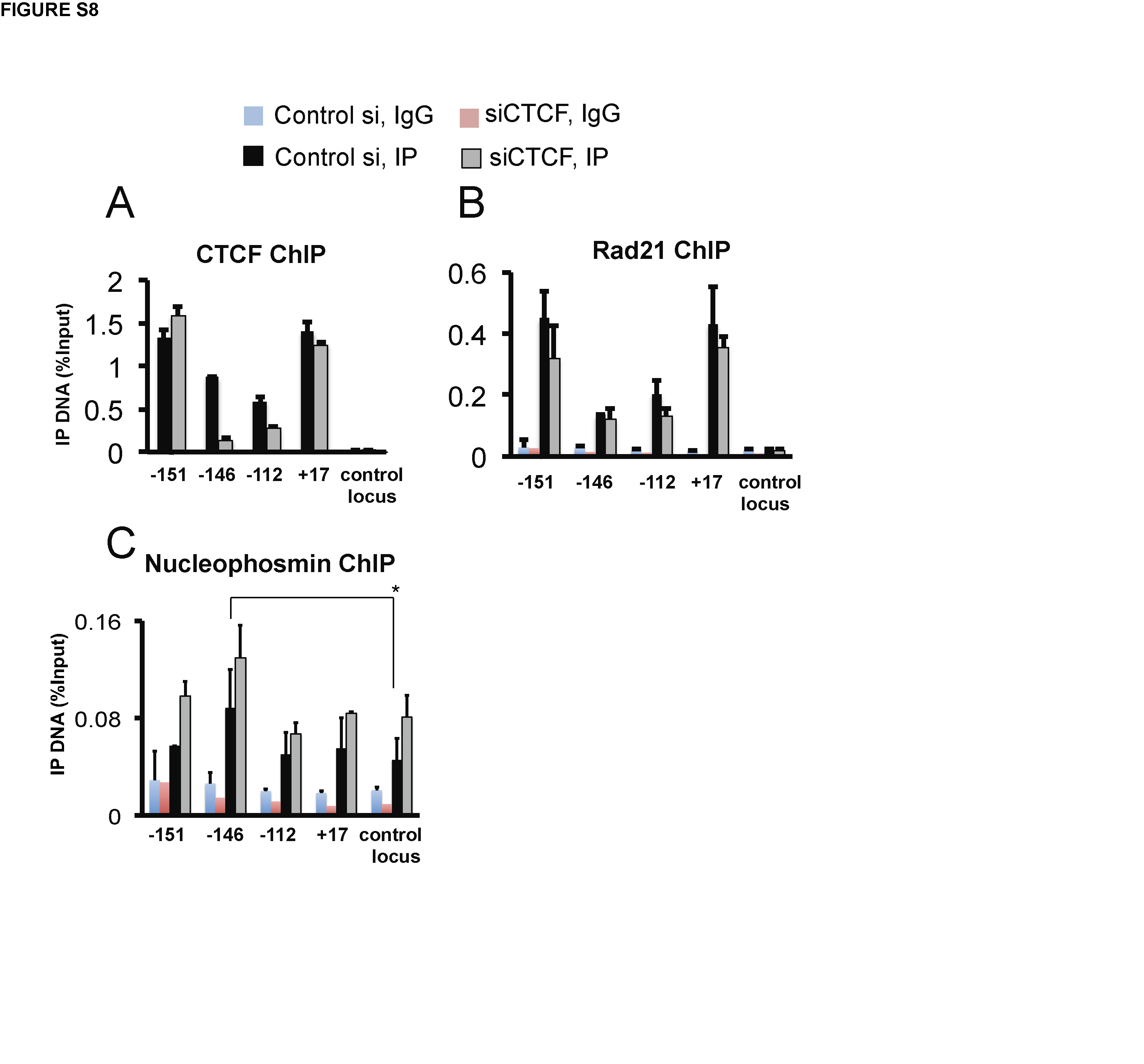

Supplement: Figure S8 — ChIP analyses of protein binding to the NANOG locus in H9 hESCs when transfected with control si or siCTCF. Statistical significance in panel C depicts fold enrichment of nucleophosmin at –146 over the negative control region. (TIF) [file pone.0042424.s008.tif]

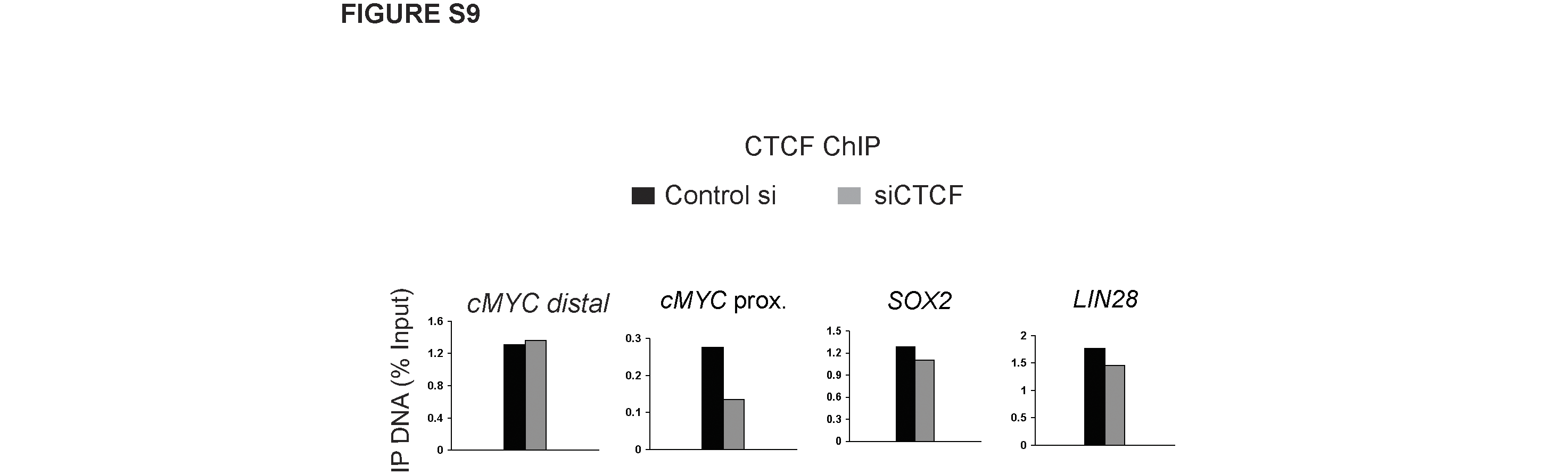

Supplement: Figure S9 — ChIP analyses of CTCF binding at indicated loci in H9 hESCs when transfected with control si or siCTCF. (TIF) [file pone.0042424.s009.tif]
